# Lack of functional alpha-lactalbumin prevents involution in Cape fur seals and identifies the protein as an apoptotic milk factor in mammary gland involution

**DOI:** 10.1186/1741-7007-6-48

**Published:** 2008-11-06

**Authors:** Julie A Sharp, Christophe Lefèvre, Kevin R Nicholas

**Affiliations:** 1 CRC for Innovative Dairy Products, Department of Zoology, University of Melbourne, VIC 3010, Australia; 2 Institute of Technology and Research Innovation, Deakin University, Geelong VIC 3214, Australia; 3 Victorian Bioinformatics Consortium, Monash University, Clayton VIC 3080, Australia

## Abstract

**Background:**

The mammary gland undergoes a sophisticated programme of developmental changes during pregnancy/lactation. However, little is known about processes involving initiation of apoptosis at involution following weaning. We used fur seals as models to study the molecular process of involution as these animals display a unique mammary gland phenotype. Fur seals have long lactation periods whereby mothers cycle between secreting copious quantities of milk for 2 to 3 days suckling pups on land, with trips to sea alone to forage for up to 23 days during which time mammary glands remain active without initiating apoptosis/involution.

**Results:**

We show the molecular basis by which alpha-lactalbumin (LALBA), a secreted milk protein, is absent in Cape fur seals and demonstrate an apoptotic function for LALBA when exposed to mammary cells.

**Conclusion:**

We propose that apoptosis does not occur in fur seal mammary glands due to lack of LALBA in fur seal milk, allowing evasion of involution during a foraging trip. Our work identifies LALBA as a milk factor that feeds back on the mammary gland to regulate involution.

## Background

The mammary gland represents one of the most dramatic examples of physiological development. The massive changes of form and function of mammary glands over the life span of a female are characterized by extreme changes in cell proliferation, differentiation, secretion and death, which accompanies pregnancy, lactation and involution upon weaning. While milk is sucked from the mammary gland it provides nutrition and immunity to the young. However, upon milk stasis, due to absence of sucking at weaning, the mammary gland regresses and is remodelled by a process known as involution, which cleanses the gland and returns it to a virgin-like state. Although the mammary gland appears vastly regulated it is also highly susceptible to cancer, with mortality associated with breast cancer rating amongst the highest causes of death for women in the western world.

The study of apoptosis in the mammary gland during involution is important for understanding both the normal biology of post-natal regression and the events leading to mammary gland tumorigenesis. Interestingly, some mammals have modified their lactation cycle in order to accommodate and adapt to extreme environmental pressures. Animals such as otariid seals (fur seals and sea lions) exhibit an unusual lactation phenotype [1] which differs from other members of the Pinnipedia family and other mammals. These animals display resistance to mammary gland apoptosis and involution after cessation of sucking, and provide a unique opportunity to investigate aspects of mammary gland physiology that are present but not readily apparent in other species.

The three families of Pinnipeds, comprising Phocids (true seals), Odobenids (walrus), and Otariids (sea lions, fur seals) evolved from a carnivorous ancestor around 25 million years ago and diverged during the middle Miocene (10 million years ago) [2]. Each family adopted different approaches to lactation. Phocid seals evolved large sizes to reduce heat loss and risk of predation and increase body reserves. This enabled them to adopt a 'fasting strategy' of lactation [3] whereby amassed body reserves of stored nutrients facilitate fasting on land during continuous milk production over relatively short periods (4 to 42 days, depending on the species).

In contrast, ancestral otariid seals retained smaller body sizes and insulating fur, and bred at rockeries to gain proximity to local prey resources adopting a 'foraging lactation' strategy [1]. The small size of otariid seals made it necessary to feed during lactation in order to replenished body stores required to continue milk production. Reduced prey availability led to lengthening of the lactation period (4 to > 12 months) and otariid seals began exploiting resources farther off shore, increasing the duration but reducing frequency of foraging trips during lactation. The current-day otariid seals produce milk with no detectable lactose [4,5] and have adopted a lactation strategy which is characterized by alternation between periods of several days of copious milk production on shore and extended periods of maternal foraging at sea [1]. Intersuckling intervals have been recorded in otariid seals of up to 23 days and are among the longest ever recorded for a mammal [1]. The need to increase duration of foraging trips due to distant foraging grounds during lactation has selected for an adapted otariid mammary gland, which remains functional despite sustained interruptions in suckling activity.

For other mammals, accumulation of milk in the mammary gland due to cessation of sucking by the young allows factors present within milk to regulate mammary epithelium, causing downregulation of milk protein gene expression, followed by involution via apoptotic cell loss [6]. During long periods at sea, in the absence of sucking, fur seal mammary glands have been recorded to produce 80% less milk than when lactating on land [7], and milk protein gene expression decreases [8], aspects which are common with cessation of sucking in other mammalian species and characteristic of the initiation of involution [9]. In other mammals these events are rapidly followed by involution comprising apoptotic mammary gland cell death [9], but the fur seal mammary gland does not undergo involution at this time [8] and remains active in readiness for return to land to continue nursing the young. The fur seal mammary gland evades the effects of engorgement by downregulating milk protein production; however, milk still remains in the gland. Therefore these animals display resistance to mammary gland apoptosis and involution following cessation of suckling and provide a unique opportunity to investigate aspects of mammary gland physiology associated with involution. There are many examples of genetic rearrangements that show strong associations with specific phenotypes that have important evolutionary consequences (see [10] for examples). In many cases a gene required for the development of a specific trait in one species shows a difference in expression in other species that correlates with a different trait [10]. These relationships provide causal evidence of plausibility.

Here we show the alpha-lactalbumin gene (*LALBA*), which normally encodes a milk protein involved in lactose synthesis, has undergone a number of *cis*-acting mutations, which render the protein absent in otariids. We show that LALBA causes apoptosis of mouse and human mammary epithelial cell lines and fur seal primary mammary cells. We propose that lactose synthesis and mammary gland involution does not normally occur in fur seal mammary glands due to absence of LALBA in fur seal milk and show that previous predictions based solely on isolation of cDNA in otariid mammary glands [11] are incorrect. We discuss and present functional evidence that directly relates to the different lactation phenotypes of otariid and phocid seals.

## Results

### *LALBA *gene expression in the fur seal mammary gland

Affymetrix canine arrays were used to examine the pattern of gene expression in mammary tissue from Cape fur seals (*Arctocephalus pusillus*) during pregnancy, on-shore lactation and off-shore lactation. These results were compared with microarray analysis patterns of genes expressed in human (*Homo sapiens*), cow (*Bos taurus*) and mouse (*Mus musculus*) mammary gland during pregnancy and lactation. Using this approach candidate apoptosis genes were examined for significant differences in expression, and unusually low levels of a major milk protein gene, *LALBA*, were observed in mammary glands of lactating Cape fur seals.

**Figure 1:**
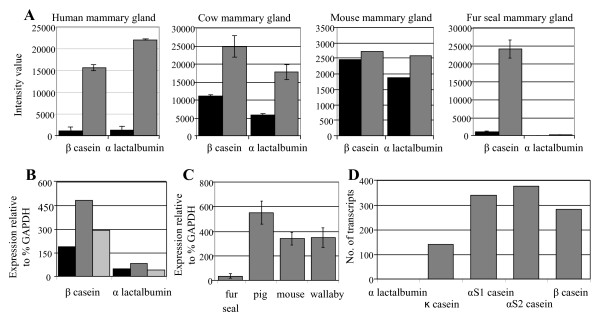
**LALBA comparative expression**. (**A**) Affymetrix analysis of *LALBA *and *β-casein *transcripts in human, cow, mouse and Cape fur seal mammary glands during pregnancy (black bar) and lactation (grey bar). (**B**) RT-PCR quantitative analysis of *LALBA *and *β-casein *expression in Cape fur seal mammary glands during pregnancy (black bar), lactation on-shore (dark grey bar) and lactation off-shore (light grey bar). (**C**) Interspecies RT-PCR quantitative analysis of *LALBA *expression levels in mammary glands of Cape fur seal, pig, mouse and wallaby during peak lactation. (**D**) Milk protein gene transcripts (from a total of 11,232 ESTs) sequenced from Cape fur seal lactating mammary gland. Standard errors are shown.

In human, cow and mouse lactation cycles, *LALBA *expression levels were found to be similar to that of other milk protein genes (146%, 68% and 96% expression relative to *β-casein*) and levels of expression of both these milk protein genes increased dramatically with onset of lactation (Figure 1A). In Cape fur seal lactation, expression of *LALBA *was observed to only increase slightly from pregnancy to lactation and was expressed at only 0.8% of the level of the *β-casein *gene (Figure 1A). Due to differences in probe sets on the Affymetrix chips, the observed low level of Cape fur seal *LALBA *expression relative to *β-casein *expression, and the lack of *LALBA *induction from pregnancy to lactation, was validated by RT-PCR using RNA extracted from mammary glands of on-shore, off-shore and pregnant seals (Figure 1B). Interspecies comparison therefore revealed Cape fur seal *LALBA *was expressed at 0.5%, 1.1% and 0.8% of the levels observed in human, cow and mouse mammary glands, respectively, during lactation.

Further analysis was performed by RT-PCR using RNA extracted from lactating mammary gland tissue from pig, mouse and wallabies using *LALBA *primers specific for each species. The wallaby was included in this analysis due to the absence of lactose in wallaby milk during peak lactation. Compared with pigs, mice and wallabies, Cape fur seals were found to have a 10 to 100-fold reduction in *LALBA *expression during lactation (Figure 1C).

In order to determine the abundance of *LALBA *transcripts relative to all other transcripts within the lactating fur seal mammary gland we undertook a large-scale global transcriptome analysis. 11,232 EST clones from a Cape fur seal lactating mammary gland were sequenced and annotated. A single *LALBA *gene transcript failed to be detected, whereas mRNAs representing other milk protein genes *κ-casein, β-casein, casein αS1-casein *and *αS2-casein *were detected at high levels (Figure 1D).

### Comparative promoter analysis

**Figure 2:**
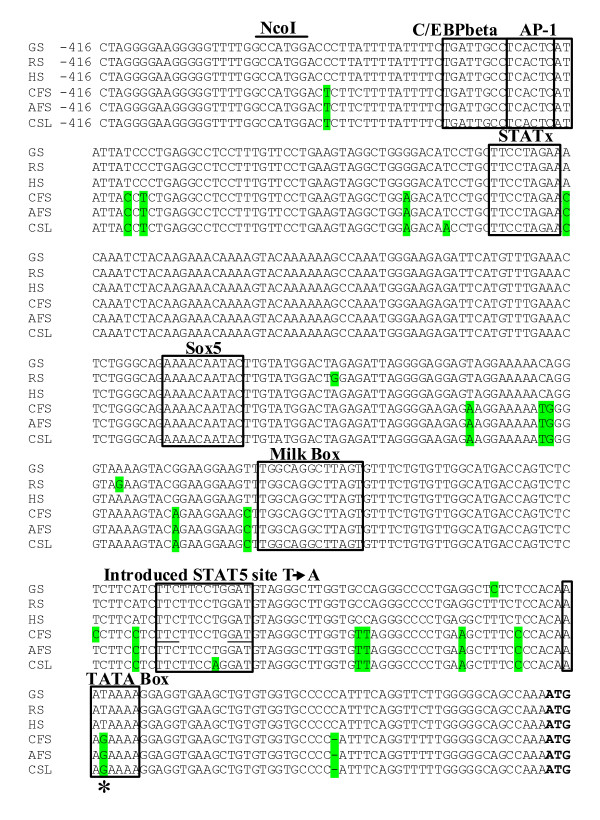
**Comparative analysis of the Pinnipedia LALBA promoter**. Comparison of nucleotide sequences of the immediate upstream promoter regions of the pinniped *LALBA *gene shows very strong conservation of nucleotide sequences among species. Divergent bases are shaded. Dashes represent gaps in the alignment. Nucleotide positions are shown relative to the start of translation (BOLD). A number of predicted binding sites are indicated. The 'milk box' [RGAAGRAA(N)TGGACAGAAATCAA(CG)TTTCTA] previously identified in *LALBA *and *casein *promoter sequences were also present at an identical position in the pinniped *LALBA *genes. The base involved in the Cape fur seal G to T mutagenesis to correct the predicted TATA box from AAGAAA to AATAA is shown by an asterisk, and bases involved in creation of a STAT5 site by converting CAAn_6_GAT to CAAn_6_GAA via mutagenesis in harbour seal and Cape fur seal are underlined. GS: grey seal, RS: ringed seal, HS: harbour seal, CFS: cape fur seal, AFS: Antarctic fur seal, CSL: California sea lion.

Comparative otariid and phocid *LALBA *promoter analysis and gene expression studies were undertaken to elucidate the differences between *LALBA *expression in these highly related species with distinctly different lactation phenotypes. Genomic DNA upstream from the untranslated region of the *LALBA *gene of three otariid seals, Cape fur seal, California sea lion (*Zalophus californianus*) and Antarctic fur seal (*A. gazella*,) was amplified, sequenced and compared with three phocid seals; grey seal (*Halichoerus grypus*), ringed seal (*Pusa hispida*) and harbour seal (*Phoca vitulina*). Analysis of a 718 bp region of the immediate proximal promoter for all seal species showed 96.78 to 97.21% sequence identity, suggesting high level of conservation. However, the TATA box, a conserved DNA sequence which initiates transcription in many genes [12], and previously defined for the *LALBA *gene in guinea pigs at positions -60 to -57 (relative to the start of transcription) [13] was altered in all three otariid seals. All three otariid species displayed a T-G transversion within the third position of the TATA box, creating an AAGAAA sequence (Figure 2). The *LALBA *TATA box in many other mammalian species comprises the sequence AATAAA, with the exception of mouse and human which comprise CATAAA and TATAAA respectively (Table 1).

**Table 1:** Interspecies comparison of LALBA TATA box sequences

Species	TATA box *	Efficiency†	Accession
Cape fur seal ‡	-58 AAGAAAA	1.2–1.5%	EU295506
Sea lion ‡	-58 AAGAAAA	1.2 – 1.5%	EU295508
Antarctic fur seal‡	-58 AAGAAAA	1.2 – 1.5%	EU295507
Grey seal^§^	-59 AATAAAA	60 – 75%	EU2955011
Harbour seal^§^	-59 AATAAAA	60 – 75%	EU295509
Ringed seal^§^	-59 AATAAAA	60 – 75%	EU2955010
Canine	-59 AATAAAA	60 – 75%	NC006609
Human	-56 TATAAAA	100 – 172%	X05153
Bovine	-59 AATAAAA	60 – 75%	M90645
Sheep	-59 AATAAAA	60 – 75%	AB040058
Camel	-53 AATAAAA	60 – 75%	AJ409281
Guinea pig	-59 AATAAAA	60 – 75%	Y00726
Goat	-59 AATAAAA	60 – 75%	M63868
Pig	-60 AATAAAA	60 – 75%	L31945
Rabbit	-59 AATAAAA	60 – 75%	AF123893
Water Buffalo	-59 AATAAAA	60 – 75%	F194373
Domestic Yak	-59 AATAAAA	60 – 75%	AF194372
Mouse	-57 CATAAAA	60 – 75%	M87863
Rat	-56 AATAAAA	60 – 75%	X00461

* numbers represent base pairs relative to start of translation

^† ^prediction based on studies by Wobbe and Struhl [12] and expressed as a percentage of the consensus TATAAAG

‡ Otariid seal

^§ ^Phocid seal

### *In vitro *expression analysis

A direct comparison between otariid and phocid *LALBA *gene expression levels was assayed by use of reporter gene constructs. This was performed in order to determine if low levels of Cape fur seal *LALBA *transcript detection were due to low levels of *LALBA *gene transcription or low *LALBA *transcript stability.

**Figure 3:**
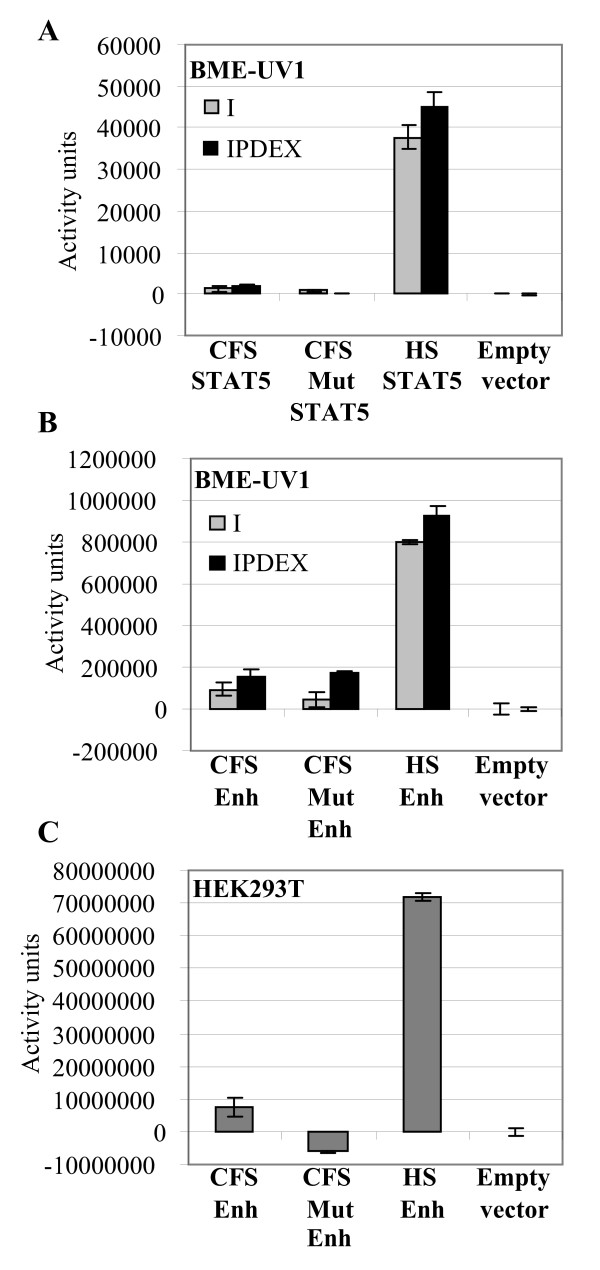
***LALBA *reporter gene expression**. **(A) **Cape fur seal (CFS), Cape fur seal TATA box mutation (CFSmut) and harbour seal (HS) reporter gene *LALBA *expression (718 bp promoter) in the presence of an introduced STAT5 site in BME-UV1 cells in the presence of insulin (I) or insulin/prolactin/dexamethazone (IPDEX) induction. **(B) **Reporter gene *LALBA *expression (398 bp promoter) in the presence of a pGL3 enhancer site in BME-UV1 and **(C) **HEK293T cells. Statistical analysis showed HS reporter gene expression in BME-UV1 cells is significantly higher than CFS in a STAT5 background (*P *= 0.038) and also in an Enhancer background in BME-UV1 cells (*P *= 0.000) and HEK239T cells (*P *= 0.010). Experiments were performed in duplicate and repeated at least twice. Standard errors are shown.

Regions of genomic DNA containing *LALBA *promoters and translation initiation codons (ATG) of an otariid seal (Cape fur seal) and a phocid seal (harbour seal) were ligated into to a luciferase reporter gene. For both species the immediate -718 bp to 3 bp promoter fragment was cloned in front of a luciferase gene within a basic vector (pGL3) and promoter activity was measured by transiently transecting plasmids into a bovine mammary epithelial cell line (BME-UV1) and a human embryonic kidney cell line (HEK293T) and measuring luciferase activity under a variety of conditions. BME-UV1 cells, which have previously been shown to induce expression of milk protein gene promoters in the presence of lactogenic hormones [14], were used to examine both basal and inducible *LALBA *expression levels, while HEK293T cells were used to measure *LALBA *expression in the presence of an introduced enhancer element, measuring the capacity of the TATA box to initiate transcription. In BME-UV1 cells, significant expression levels failed to be detected for both seal species so a STAT5 site was subsequently introduced by *in vitro *mutagenesis (Figure 2) to enhance *LALBA *expression, as previously described for the mouse *LALBA *promoter [15]. With the addition of the STAT5 site, the harbour seal *LALBA *reporter constructs showed significantly higher levels of expression in BME-UV1 cells under non-induced (insulin) and induced conditions (insulin, prolactin, dexamethazone), compared with the Cape fur seal *LALBA *reporter constructs (*P *= 0.038) (Figure 3A), similar to that shown for the mouse *LALBA *promoter in the presence of an introduced SAT5 site [15]. The influence of the otariid TATA box (AAGAAA) on gene transcription was analysed by correcting the Cape fur seal TATA box (AAGAAA to AATAAA) using *in vitro *mutagenesis to introduce a T into the G position. The *in vitro *mutated Cape fur seal TATA box (AATAAA) alone failed to significantly increase levels of transcription compared with the wild-type Cape fur seal (AAGAAA) promoter construct in the presence of the introduced STAT5 site. Each promoter was also assayed in BME-UV1 cells using an enhancer sequence (pGL3-Enhancer) to elevate basal transcription levels. Again the harbour seal *LALBA *reporter constructs showed significantly higher levels of expression compared with the Cape fur seal *LALBA *reporter constructs (*P *= 0.000) and the *in vitro *mutated Cape fur seal TATA box (AATAAA) alone failed to significantly increase levels of transcription compared with the wild-type Cape fur seal (AAGAAA) promoter construct (Figure 3B). The *LALBA::luciferase *constructs were also tested in HEK293T cells with the same results (Figure 3C).

Use of different host cell lines eliminated the probability that the observed expression pattern was not cell-line specific. These results showed that the low level of Cape fur seal *LALBA *expression observed occurred at the level of transcription and was not due to low *LALBA *transcript stability. These results also showed that the Cape fur seal *LALBA *TATA mutation alone is not responsible for the low level of expression observed (Figure 3A to 3C).

### Identification of LALBA transcripts

**Figure 4:**
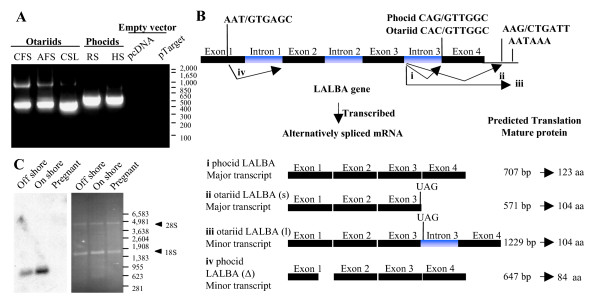
***LALBA *transcription**. (**A**) RT-PCR shows equal expression of each *LALBA *transgene with one major mRNA transcript for each species. Markers are shown (bp). **(B) **Otariid and phocid *LALBA *gene structure and mRNA splice variants. The *LALBA *gene comprises four exons that undergo alternative splicing. Identified transcript variants (indicated by i, ii, iii and iv) comprise (i) a correctly spliced phocid *LALBA *transcript; (ii) an otariid *LALBA *transcript which is formed by use of a 3' splice site in the *LALBA *3'UTR (shown) prior to the *LALBA *termination codon (shown) causing a loss of exon 4; (iii) an otariid *LALBA *transcript which contains intron 3 caused by lack of splicing due to a mutation in the 3' splice site of intron 3 (mutation shown for otariid *LALBA *gene compared with correct splice sequence in phocid *LALBA *gene); and (iv) a transcript which is formed by use of a 5' splice site within exon 1 causing a 60 bp deletion of exon 1, shown here for phocid *LALBA*. The Otariid *LALBA *gene also has this 5' splice site (transcripts not shown here). **(C) **Northern blot (left panel) confirms one major *LALBA *transcript is detected in the Cape fur seal mammary gland. Right panel shows corresponding ethidium bromide-stained mRNA prior to blotting.

The *LALBA *gene was previously found to contain a mutation in the 3' splice site of intron 3 causing incorrect splicing of the third exon giving rise to two transcripts [11]. Due to the very low level of expression of otariid *LALBA *it is difficult to identify the number and full range of *LALBA *transcripts. In order to determine the nature and relative abundance of different *LALBA *splice variants of the otariid seals (Cape fur seal, California sea lion and Antarctic fur seal) and phocid seals (ringed seal and harbour seal), *LALBA *genomic fragments were cloned into CMV expression vectors and transfected into HEK293T cells. Genomic fragments comprised all 4 exons and 3 introns with 3'UTR. Following transfection, analysis of *LALBA *transcripts by RT-PCR and sequence analysis showed the presence of one major otariid *LALBA *transcript, corresponding to *LALBA(s)*, with the structure exon 1–2–3 and lacking exon 4, and two minor transcripts (Figure 4A). One of the two minor transcripts corresponded to *LALBA(l) *with the structure exon 1–2–3, intron 3 exon 4, while the second minor transcript *LALBA*(Δ) is due to incorrect splicing of intron 1 leading to deletion of the final 60 bp in intron 1. This deletion is caused by use of a 5' splice consensus sequence (AAT/GTGAGCT) within intron 1 and is fused in frame with intron 2, causing an in-frame deletion of 20 amino acids. Analysis of phocid seal (ringed seal, common seal) *LALBA *genomic fragments revealed the presence of one major *LALBA *transcript corresponding to correctly spliced exons 1, 2, 3 and 4 and a minor transcript corresponding to *LALBA*(Δ) (Figure 4B). The presence of one major *LALBA *transcript within the Cape fur seal mammary gland was confirmed by northern blot analysis (Figure 4C). Expression of *LALBA *was observed to increase during lactation on-shore compared with *LALBA *expression off-shore.

### Otariid *LALBA *transcripts are not translated into protein

**Figure 5:**
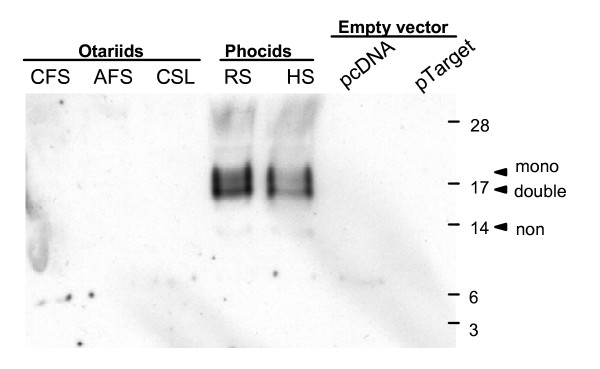
***LALBA *translation**. LALBA immunoreactive protein from supernatants, collected 24 hours after transfection of expression constructs shows non-glycosylated (non), mono-glycosylated (mono) and double glycosylated (double) forms of phocid LALBA, while otariid LALBA was not detected. Markers are shown (kD).

Previous predictions based on *in silico *translations of otariid cDNAs suggested the protein is truncated, removing the final 19 residues [11]. Conditioned media from cells transfected with otariid and phocid *LALBA *genomic fragments was tested for presence of secreted LALBA at 24 and 72 hours by western blot using a polyclonal anti-bovine LALBA antibody. Although expression levels of otariid and phocid *LALBA *transcripts were observed to be equal due to presence of the CMV promoter (Figure 5A), and equal amount of protein was loaded per lane and confirmed by coomassie blue staining of protein within the gel (data not shown), LALBA protein was absent in the conditioned media of cells expressing otariid seal (cape fur seal, California sea lion, Antarctic fur seal) *LALBA *genes, while LALBA protein was detected in the conditioned media of cells expressing phocid seal (ringed seal and harbour seal) *LALBA *genes (Figure 5). A crude approximation of phocid LALBA protein production was determined by comparison with a known bovine LALBA (bLALBA) (10 μg) standard. Using this method approximately 0.01 μg phocid LALBA/ml was estimated to be present within the conditioned media. Due to differences in immunoreactivity between phocid LALBA and bovine LALBA protein, concentrations were also calculated by estimating the amount of LALBA within the total protein sample. Phocid LALBA protein was not visible by coomassie staining of 300 μg total protein from 1 ml conditioned media. We can therefore conservatively estimate that phocid LALBA is less than 10% of the total protein within the conditioned media (0.03 mg/ml). The translation product from the minor phocid *LALBA*(Δ) transcript, predicted to be 12.2 kD, was not observed.

### Apoptotic effects of LALBA

**Figure 6:**
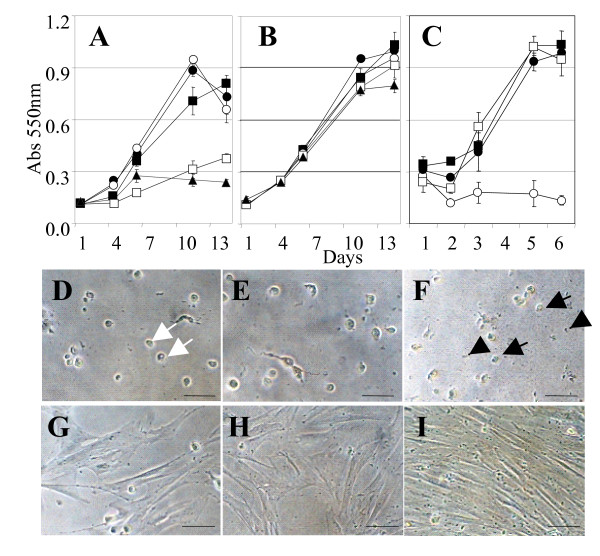
**bLALBA effects on CfsMCs**. (**A**) CfsMCs exposed to bLALBA or (**B**) BSA ●0 mg/ml, ○0.2 mg/ml, ▿0.4 mg/ml, □0.8 mg/ml, ▲1.6 mg/ml. (**C**) Loss of bLALBA activity by heat treatment or pepsin digestion ●1 mg/ml LALBA heat treated, □1 mg/ml BSA, ○1 mg/ml LALBA, ●1 mg/ml LALBA with pepsin digestion. (**D**) Morphology of CfsMCs exposed to bLALBA shows cells relinquish contact and detach from the substratum after 24 hours causing their rounding (white arrows). (**E**) After 2 days and (**F**) 5 days of bLALBA exposure cells exhibited shrinkage, loss of cell membrane integrity and membrane blebbing (black arrows) and accumulation of apoptotic bodies (black arrowheads). (**G**) CfsMCs exposed to 1 mg/ml BSA after 24 hours (**H**) 2 days and (**I**) 5 days. Scale represents 100 μm.

We investigated the death-inducing properties of bLALBA in the fur seal mammary gland by assaying LALBA apoptotic potential using Cape fur seal mammary cells (CfsMCs). These cells are isolated from mammary tissue and comprise a population of epithelial cells, myoepithelial cells and fibroblasts that have previously been shown to form mammospheres capable of expressing milk protein genes under induction by lactogenic hormones [16]. bLALBA (0.2 to 1.6 mg/ml) was added to CfsMCs and cell number was assessed by protein staining using an SRB assay. Its principle is based on the ability of the protein dye sulforhodamine B to bind electrostatically and pH-dependently on basic amino acid residues of trichloroacetic acid-fixed cells. The SRB assay has been shown to be an appropriate and sensitive assay to measure drug-induced cytotoxicity [17]. Physiologically relevant levels of bLALBA (0.6 to 1.6 mg/ml) produced a decrease in proliferation of CfsMCs in culture which was dose-dependent (Figure 6). Cell morphology was examined after exposure to LALBA to determine the health of the cells. CfsMCs exhibited rounding of cells and increased detachment from the substratum after 24 hours exposure to LALBA. After 5 days cells exhibited shrinkage, loss of membrane integrity and accumulation of apoptotic bodies characteristic of the apoptosis process [18] (Figure 6D to 6I). The decrease in cell number was therefore shown to be due to loss of cell number, not due to decreased cell proliferation of healthy cells. The bLALBA produced by Sigma was prepared from unpasteurized milk and declared to be 99% pure. In order to show that the observed effects were not due to toxins or contaminants the bLALBA preparation was heat-treated to denature the active protein (Figure 6C). Following heat treatment bLALBA lost the ability to induce cell death, suggesting the inducing agent was of protein origin. The doses of bLALBA inducing this effect were observed to be equivalent to levels of LALBA normally found in the milk of most mammals [19]. Cell death was not initiated at lower concentrations (0.2 to 0.4 mg/ml), even after an extended culture period of 14 days. We tested the ability of bLALBA fragments to induce cell death by digesting the preparation with pepsin. Similar to heat treating, the apoptotic effects of bLALBA were also reversed by pepsin digestion (Figure 6C), suggesting that LALBA is not active as peptide fractions.

**Figure 7:**
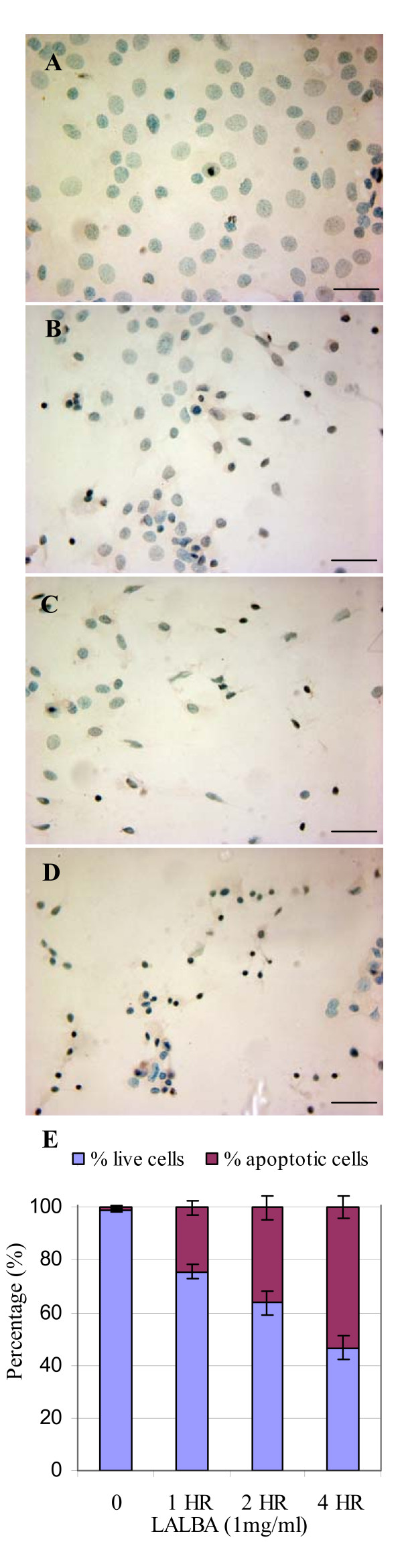
**bLALBA apoptosis rate**. Apoptotic HC11 cells (brown) are shown. (**A**) 0 mg/ml LALBA, (**B**) 1 mg/ml LALBA/1 hour, (**C**) 1 mg/ml LALBA/2 hour, and (**D**) 1 mg/ml LALBA/4 hour treatments. Scale bars represent 20 μm. (**E**) The percentage of apoptotic cells increased significantly (*P *< 0.001) for each time point versus the control.

In order to determine whether bLALBA was inducing apoptosis, as has been observed in RAW 264.7 cells [20], we treated mouse mammary epithelial cells, HC11, with LALBA and measured DNA fragmentation by Tunnel staining. Increased apoptosis was observed after 1, 2 and 4 hours following LALBA treatment (Figure 7). The number of apoptotic cells increased significantly in a time-dependent manor (*P *< 0.001).

**Figure 8:**
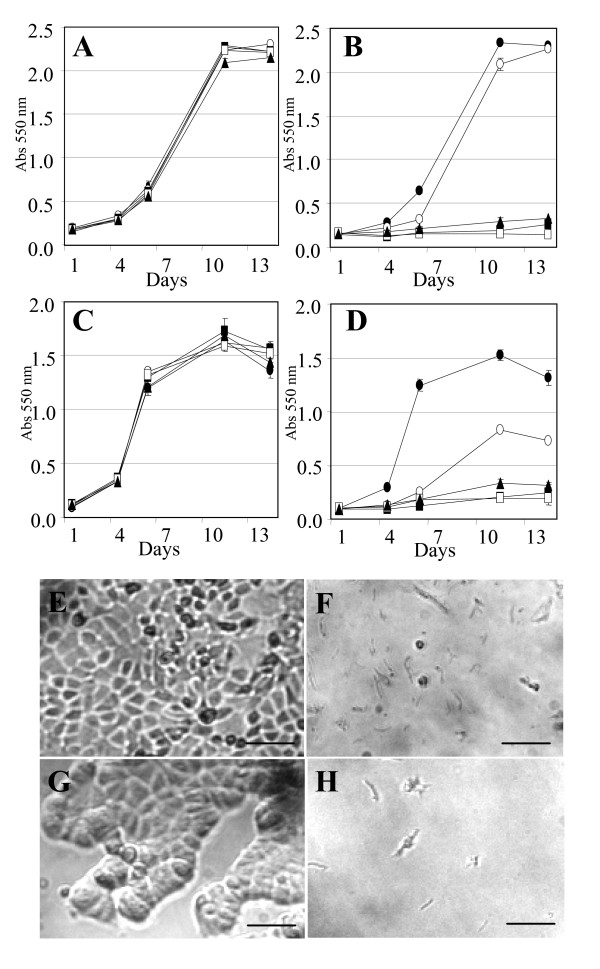
**bLALBA dose-dependent effects on HC11 and MCF-7 cells**. **(A**-**B) **HC11 and (**C **– **D**) MCF-7 cells exposed to BSA (**A **and **C**) or bLALBA (**B **and **D**). ●0 mg/ml, ○0.2 mg/ml, ■0.4 mg/ml, □0.8 mg/ml, ▲1.6 mg/ml. (**E **and **F**) Morphology of HC11 and (**G **and **H**) MCF-7 cells grown for 14 days in 1.6 mg/ml BSA (**E **and **G**) or 1.6 mg/ml bLALBA (**F **and **H**). Scale bars represent 100 μm.

In order to show that bLALBA induces dose-dependent apoptotic responses in other mammary epithelial cell lines, bLALBA was added in increasing concentrations to mouse mammary epithelial cells, HC11, and to the human breast cancer cell line MCF-7. Similar to CfsMCs, higher doses of bLALBA caused all cells to die, while lower does allowed survival of a small population of cells which went on to proliferate (Figure 8A to 8D). Morphological examination of HC11 and MCF-7 cells following 5 days of LALBA exposure demonstrated total loss of cellular material. Loss of membrane integrity and cell shrinkage was also observed, consistent with cell death pathways (Figure 8E to 8H).

### Characterization of apoptotic effects

**Figure 9:**
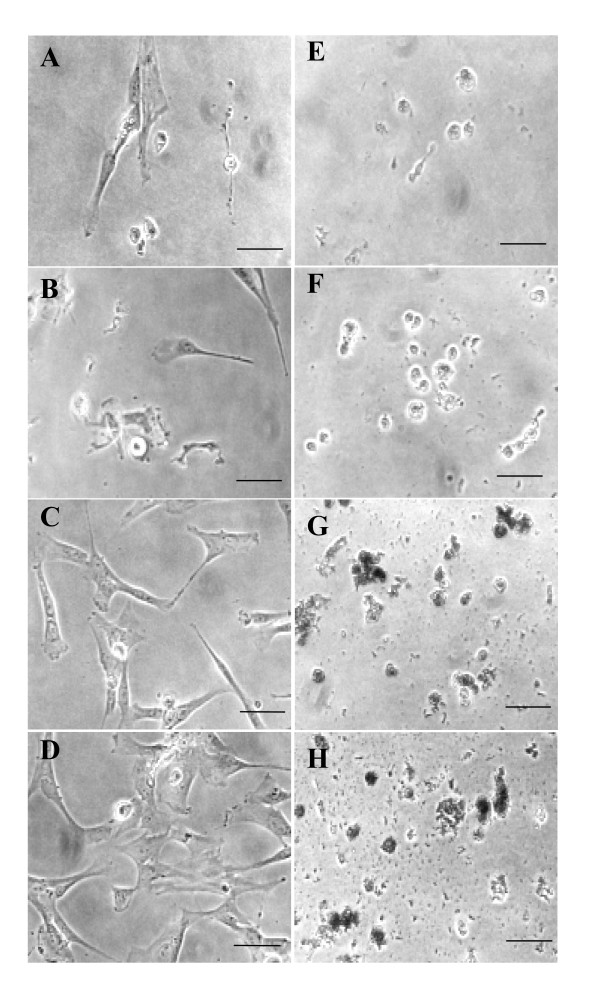
**Cape fur seal mammary cells require extended exposure of bLALBA for induction of apoptosis**. **(A **– **D**) 1 mg/ml bLALBA was added to CfsMCs for 1 hour, LALBA was then washed out and fresh media was added. (**E **– **H**) For comparison, 1 mg/ml LALBA was added to cell culture media and allowed to remain on the cells. Cells were photographed at day 1 (**A **and **E**), day 2 (**B **and **F**), day 4 (**C **and **G**) and day 5 (**D **and **H**). Scale bars represent 100 μm.

The time required for bLALBA to induce an apoptotic response was also examined by exposing cells to bLALBA for 1 and 8 hours and then removing bLALBA and replacing with fresh media. These experiments showed apoptotic effects of bLALBA were observed when cells were exposed to 8 hours of bLALBA and then removed, but apoptosis was not observed when cells were exposed for 1 hour and then removed (Figure 9).

## Discussion

Specific triggers for the process of mammary involution are unknown but autocrine feedback mechanisms have been proposed [21]. The source of this mechanism is thought to be factors in milk that interact with the mammary epithelium and trigger involution after a period of milk stasis. It was therefore proposed that fur seals, which exhibit a phenotype where involution is delayed even after very long periods of milk stasis, may lack these factors in their milk, or may lack the machinery to initiate the involution response.

It has long been established that LALBA, a major milk component, plays a central role in the mammary gland as the regulatory subunit of lactose synthase [22]. We show here that otariid (Cape fur seal) mammary glands express very low levels of *LALBA *mRNA during the lactation cycle compared with expression levels in other mammals, which show *LALBA *is one of the most highly expressed genes in the mammary gland during lactation [23]. LALBA has also recently been implicated in the induction of apoptosis of a human colon adenocarcinoma cell line [24] and RAW264.7 cells [20]. LALBA was therefore a likely candidate for further study in the involution process.

Comparison of *LALBA *expression levels between otariid and phocid seals showed otariid (Cape fur seal) *LALBA *expression levels were very low and suggested a negligible amount of transcription occurs from this promoter. The low level of otariid *LALBA *transcription was predicted to be due to an altered TATA box in otariid seals. All three otariid species displayed a T-G transversion within the third position of the TATA box creating an AAGAAA sequence. This substitution is predicted to lead to poor binding of the TATA binding protein and RNA polymerase which are necessary for transcriptional activity [25]. The consequence of a G to T substitution in the third position has previously been demonstrated, showing transcriptional activity is reduced to 2% [12]. Correction of this mutation in the Cape fur seal *LALBA *promoter failed to increase levels of transcription, suggesting that another/other mutations also play a role in preventing successful transcription of *LALBA*. Like most pseudogenes where evolutionary pressures are no longer required to prevent divergence, the otariid *LALBA *has likely undergone a number of promoter mutations, which are conserved in other lineages where *LALBA *is functional.

Analysis of *LALBA *transcript abundance showed one major transcript, *LALBA(s) *was generated for all otariid species, with *LALBA(l) *and *LALAB*(Δ) detected as minor transcripts. Interestingly, *LALAB*(Δ) was also detected in phocid species. Other species such as dogs, humans, sheep, rats, mice and pigs also have the same cryptic 5' splice sequence in exon 1, suggesting the presence of *LALAB*(Δ) in species other than seals.

Translation of otariid and phocid *LALBA *transcripts showed otariid *LALBA *mRNA failed to be translated into secreted protein. These data are consistent with earlier studies, which have failed to detect LALBA protein in milk of otariid species [26,27]. These data suggest the predicted translation of two isoforms of truncated LALBA protein (Reich and Arnould [11]) are incorrect. The authors suggest this is the mechanism for lack of lactose in fur seal milk and that lack of lactose as a major milk osmole has facilitated the fur seal lactation strategy and prevented engorgement while at sea. Contrary to this, it has been postulated elsewhere that other osmoles are present in otariid milk, such as Fuc(α1–2)Gal(β1–4)Glc, which is also found in related species of the order Carnivora [28]. These trisaccharides may provide an alternative osmotic mechanism to move water from the extracellular fluid into the milk. All these species have only small amounts or no lactose relative to oligosaccharides. Myo-inositol and free amino acids are also found at high concentrations in otariid milk [5] and exceptionally high concentrations of taurine are found in Pinnipeds [29]. Therefore, it is likely myo-inositol and taurine may play a significant role as organic osmolites. There are other examples in nature where lactose is not required for milk production. In tammar wallaby (*Macropus eugenii*) milk, carbohydrate is low and lactose is absent throughout peak lactation [30], during which time other unknown factors act as the major osmole, demonstrating that lactose is not necessary for milk production in some species. The high level of milk production by fur seals lactating on-shore ultimately requires the presence of an osmole, be it trisaccharides or organic osmolites, while the absence of engorgement in fur seal mammary glands while at sea is likely the result of reduced milk production by downregulation of milk synthesis at the transcriptional level [8].

Evidence presented here shows that bLALBA causes apoptosis of mouse and human mammary epithelial cell lines and fur seal primary mammary cells, demonstrating that although LALBA is absent in fur seal milk, the LALBA-mediated apoptotic pathway is still intact in fur seal mammary cells. It is interesting to note that all cell types in the Cape fur seal mammary population responded to bLALBA, suggesting that the apoptotic response to LALBA is not limited to epithelial cells, but also affects other cells types such as fibroblast and myoepithelial cells. The loss of LALBA apoptotic activity by heat treatment confirms the active component is of protein origin and suggests the apoptotic effects observed do not occur when the protein is fragmented by digestion with pepsin. Previously a multimeric form of LALBA (MAL), isolated from the casein fraction of human milk and formed by low pH treatment and ion-exchange, has been shown to reduce cell viability in kidney, intestine, bladder, lung cell lines, lymphocytes and thymocytes, but healthy cells were not affected [31] and mammary epithelial cells were not tested. HAMLET (human α-lactalbumin made lethal to tumour cells) [32], which is chemically treated to resemble MAL in structure, is a complex of apo-LALBA combined with oleic acid. The oleic acid co-factor binding of HAMLET is very unstable and is easily displaced by foetal calf serum (FCS). All apoptosis-inducing experiments involving HAMLET need to be performed in the absence of FCS, unlike the experiments presented here, which show LALBA inducing apoptosis in the presence of 10% FCS, suggesting that the LALBA in the current study is different in structure to HAMLET. HAMLET has been shown to localize to the nucleus where it binds to histones, disrupts chromatin structure and leads to cell death [33]. It has also been shown that negatively charged untreated LALBA can also bind to positively charged histones without the aid of oleic acid, causing aggregation [34] and suggesting that other natural forms of LALBA can induce apoptosis as observed in the current study. Indeed, untreated bLALBA has previously been found to have an apoptotic effect on cell types other than mammary cells. LALBA has previously been shown to induce apoptosis in human colon adenocarcinoma cell lines [24] and RAW264.7 cells [20] using the same bLALBA preparations from Sigma [20]. Analysis in RAW2.64.7 cells showed cells exposed to bLALBA exhibited cell shrinkage, disruption of cellular membranes, accumulation of apoptotic bodies, DNA fragmentation, an increase in sub-G1 cells, increased Annexin V expression and activation of caspase 3 [20]. These results are characteristic of the apoptotic process and show that bLALBA indeed induces a programmed cell death response via apoptosis. Similarly, we have also observed cell shrinkage, disruption of cellular membranes, accumulation of apoptotic bodies and DNA fragmentation in this study using mammary cells following exposure to bLALBA. We have also observed an increase in caspase 3 expression following exposure of HC11 cells to bLALBA for 5 hours (Brennan et al, manuscript in preparation), suggesting the same apoptotic pathway is activated in mammary cells as in RAW264.6 cells. In the current study we expressed phocid *LALBA *in HC11 cells without observing apoptosis of the host cell. Although expression of the transgene was observed to be high due to the presence of a CMV promoter, the amount of protein collected in the conditioned medium was determined to be less than that required for effective LALBA-induced apoptosis. In addition, stably transfected cells have been derived which express bLALBA which are also not detrimentally affected [35]; we suggest that these cell lines are also not capable of producing the high concentration of LALBA (0.2 to 1.6 mg/ml) necessary for induction of apoptosis.

Our data suggest that low levels of LALBA may not cause apoptosis in the *in vivo *epithelium. It could be postulated that LALBA acts only at a critical concentration and requires a specific amount of time in contact with the cell, or undergoes a conformational change during milk stasis in order to elicit its apoptotic potential. Involution only occurs following milk stasis and it has been previously demonstrated that LALBA concentrations increase in milk during mammary gland involution, while other milk proteins show decreases in concentration [36]. LALBA may cause limited apoptosis in the *in vivo *epithelium during lactation, as seen by decreased milk production in cows as lactation proceeds [37]. This gradual reduction in milk production over the lactation period has been referred to as 'gradual involution'. In fur seals milk production does not decline as in other mammals, but has been shown to increase as the pup grows larger. This is presumed to sustain the nutritional needs of a growing pup. The lack of LALBA in fur seals may not only allow these mammals to circumvent involution, but may also aid in avoiding gradual involution observed in other lactating mammals.

We suggest that exposure of mammary epithelial cells to LALBA may be a mechanism for mammary gland involution during milk stasis at weaning, a time when LALBA levels increase in the milk. To support this we have presented a molecular analysis of a relevant lactation model, the fur seal, which avoids involution in the presence of milk stasis and showed that these animals do not produce LALBA in their milk. In addition we have shown that mammary epithelial cells, when exposed to similar LALBA concentrations to those found in milk, undergo an apoptotic response.

## Conclusion

We propose that mammary gland involution does not normally occur in fur seal mammary glands due to absence of LALBA in fur seal milk and not due to the presence of a truncated LALBA protein as previously reported [11]. The absence of LALBA has therefore facilitated the divergence of the fur seal species during evolution and enabled this species to adopt a unique lactation phenotype to exploit their environment. This hypothesis is supported by previous observations of *LALBA*-deficient mice [38]. Milk of *LALBA-*deficient mice [38] is high in lipid and protein but very low in lactose; however, mothers are not capable of feeding their young as milk is highly viscous and cannot be removed by normal suckling. Mice, unlike fur seals, rely on lactose as their major osmole and therefore milk in this mouse was devoid of water, making sucking-induced removal of milk from the mammary gland impossible. After 5 days of lactation, alveoli of *LALBA*-deficient mouse mammary glands were distended and so engorged with milk that tight junctions were compromised and milk was observed to move from the alveolar space and enter into the surrounding mammary tissue. However, there was no evidence of apoptosis or involution which would normally occur by this time in the absence of milk removal [39]. The absence of apoptosis and involution in the *LALBA*-deficient mouse is consistent with absence of apoptosis and involution in the *LALBA*-deficient otariid seal.

The tammar wallaby is another model species with an unusual *LALBA *phenotype. As discussed previously, wallaby milk during peak lactation is devoid of lactose; however, LALBA is secreted in wallaby milk at similar levels to other mammals throughout lactation [30]. Examination of expression levels of wallaby *LALBA *in the current study showed similar levels to other mammals such as pig and mice during peak lactation. This shows that wallabies have uncoupled secretion of LALBA and lactose production. In contrast to fur seals, wallabies do not have an interrupted pattern of lactation and do not have long periods of non-suckling activity, so we predict that continued secretion of LALBA during wallaby lactation is required solely for rapid initiation of mammary gland involution at weaning.

Here we show that LALBA acts an as apoptotic inducer of mammary epithelial cells and we correlate the lack of LALBA with the absence of apoptosis in fur seal mammary glands. This study has shown how lack of LALBA has had important evolutionary consequences leading to the phenotypic trait of foraging lactation in fur seals that is different from the fasting lactation in phocid seals, and by doing so identifies LALBA as a likely candidate trigger for involution in the mammary gland.

## Methods

### RNA preparation

RNA was isolated (QIAGEN RNeasy Lipid Kit, Australia) from Cape fur seal, wallaby, pig and mouse mammary tissue or from cells derived from California sea lion and Antarctic fur seal milk during peak lactation, human milk and colostrum at 24 hours prior to birth and at 3 and 4 months peak lactation, or HEK293T cells. mRNA purification was performed by NucleoSpin kit (BD Biosciences).

### Microarray analysis

RNA isolated from Cape fur seal mammary glands during pregnancy (*n *= 2), on-shore lactation (*n *= 2) and offshore foraging (*n *= 1) or human during pregnancy (*n *= 2) and lactation (*n *= 2) was used for gene profiling analysis. Labelling of RNA and microarray processing was contracted to the Australian Genome Research Facility (Melbourne, Australia) using Canine genome 2.0 or Human genome Affymetrix genechips. Cow Affymetrix results were kindly provided by Dr PA Sheehy, Faculty of Veterinary Science, University of Sydney, NSW, Australia. Data was normalized using RMA methods in Bioconductor [40]. Mouse Affymetrix results were obtained from [39].

### Off-shore Cape fur seal EST library

mRNA (1 μg) was used to construct a cDNA library using Creator SMART cDNA Library Construction Kit (BD Biosciences). A total of 11,232 ESTs were sequenced by the Australian Genome Research Facility, Brisbane, Australia.

### RT-PCR

Total RNA (0.1 μg) was used to create cDNA. 0.2 μl cDNA was used in PCR with 20, 25 and 30 cycles of amplification to determine the linear range. Each PCR was performed at least twice. See additional file 1: supplementary Table 1 for primer sequences. Images of PCR products resolved in ethidium bromide-stained agarose gels were visualized using UVP BIODoc-it System (Pathtech, Australia) and saved by PCTV Vision software. Quantification was performed by densitometry using ImageJ software (NIH). Expression levels were estimated by normalizing expression against GAPDH.

### Northern blotting

mRNA (1 μg) was electrophoresed in 1.2% agarose gels, blotted to Zeta Probe and hybridized with a 32P-labelled Cape fur seal *LALBA *cDNA probe (550 bp).

### Genomic LALBA expression constructs

DNA was extracted from Cape fur seal liver and California sea lion and Antarctic fur seal milk. DNA from ringed seal, grey seal and harbour seal were gifts from Dr P Johnson (Ohio University, USA), Dr M Walton (University of St. Andrews, Scotland) and Dr N Lehman (Portland State University, USA), respectively. LALBA coding sequences were amplified using specific primers spanning the ATG of exon 1 and 3' UTR (Additional file 1: supplementary Table 1). Amplified products were cloned into pTarget (Promega) and sequenced. Fragments in incorrect orientation were digested using *Not*I and *Xho*I restriction sites flanking the *LALBA *fragment and were cloned into pcDNA3.1 (Promega) and sequenced. Both vectors contain CMV promoters and all constructs contained a Kozak sequence A/GTGATTATGA. Cape fur seal, Antarctic fur seal, and harbour seal *LALBA *were cloned into pTarget, while ringed seal and sea lion DNA were cloned into pcDNA 3.1.

### Reporter constructs

*LALBA *5' promoter sequences were amplified using primers corresponding to -770 bp to -750 bp and -12 bp to +3 bp containing an *Nco*I restriction site. Amplified DNA was cloned into pGemt-easy (Promega) and sequenced. Mutagenesis of TATA box and STAT5 sites was performed by PCR and sequenced. Plasmids were digested with *Nco*I/*Sac*I using sites within the vector and 718 bp and 398 bp fragments were subcloned into pGL3-basic and pGL3-enhancer respectively and sequenced. See additional file 1: supplementary Table 1 for primers.

### Preparation of Cape fur seal mammary cells

Mammary tissue was obtained from one Cape fur seal, which was confirmed to be pregnant and non-lactating from inspection of the uterus and mammary glands, respectively. Tissue was immediately transferred to 1× Hanks' Balanced Salt Solution (HBSS) (Sigma Aldridge, Sydney, Australia) with 10 μl/ml penicillin/streptomycin (Gibco, USA) and 2.5 μg/ml Fungizone (Gibco, USA) on ice and transported back to the laboratory for enzymatic digestion to harvest mammary epithelial cells. Mammary tissue was dissected free from fat, weighed, sliced finely and digested with collagenase and hyaluronidase (25 g tissue per 100 ml media) at 37°C for 4 hours. Cells were harvested by filtration (Nalgene filter, 53 μm and 200 μm mesh). The suspension was centrifuged 80 g/5 minutes, pellets were washed twice with HBSS. Cell suspensions were centrifuged, resuspended in M199 and 90% FCS/10% DMSO (DMSO-Sigma, Sydney, Australia), and frozen at a density of ~2 × 10^7 ^cells/ml.

Cape fur seal mammary cells were cultured in either 25 or 80 cm^2 ^culture flasks in 5 ml or 10 ml respectively of M199/Hams/Hepes media with 1 μg/ml cortisol, 10 ng/ml EGF, 1 μg/ml insulin supplemented with 20% horse serum and 5% foetal bovine serum. The cells were passaged (up to three times) when confluent using 0.1% trypsin-versine solution in phosphate buffered saline (Sigma-Aldrich, Sydney, Australia).

### Cell culture

HEK293T and MCF-7 were grown in DMEM/10% FCS, HC11 cells were grown in M199/HAMS/HEPES/10% FCS, CfsMCs were grown in M199/HAMS/HEPES/20% horse serum/5% FCS with 1 μg/ml cortisol, 10 ng/ml EGF, 1 μg/ml insulin (I) and BME-UV1 cells were grown in 50% DMEM/30% RMPI/20% NCTC-135/5% FCS with 1.0% lactose, 0.1% lactalbumin hydrolysate, 1.2 mM glutathione, 5 μg transferrin/ml, 10 μg L-ascorbic acid/ml. For induction, BME-UV1 media was supplemented with I (1 μg/ml), prolactin (P) (1 μg/ml), dexamethazone (Dex) (1 μM).

### Transfection

Prior to transfection BME-UV1 cells were induced for 24 hours with I or I, P, Dex. Transfection was performed using lipofectamine (Invitrogen). Reporter constructs were co-transfected with β-glycosidase vector. Induction or growth media was replaced after 6 hours and incubated for 2 days. For *LALBA *transcript and protein analysis media was changed to DMEM/2% FCS and incubated for 72 hours. Media was collected at 24 and 72 hours. After 72 hours RNA was isolated.

### Assays

Luciferase assays were performed using Luciferase Assay reagent (Promega, USA) and β-glycosidase assays were performed according to the β-galactosidase Enzyme and Assay System with Reporter Lysis Buffer Kit (Promega, USA). Luciferase expression values were normalized by comparison with β-glycosidase activity. Each experiment was performed in duplicate and repeated at least twice.

### Western blot

1 ml of conditioned media (24 and 72 hours) was washed free from salts by use of Amicon ultra 1 kD columns (Millipore). Supernatants were concentrated and resuspended in 50 μl dH_2_O and assayed for protein by BCA assay (Pierce, Rockford, IL). 300 μg protein (~50 μl) was separated on 15% SDS-PAGE gel and transferred to PVDF membrane. Coomassie staining of the gel confirmed an equal amount of protein was loaded per sample. Immunoblotting was conducted by using rabbit polyclonal antibodies specific for LALBA, detected with a goat anti-rabbit HRP-conjugated antibody (Pierce), and visualized using SuperSignal chemiluminescent substrate (Pierce, Rockford, IL). The experiment was performed in triplicate using two time points per sample.

### Proliferation assay and cell morphology

Cells were plated (2000 cells/well) in 96-well plates in growth media supplemented with 10% FCS. After one day bLALBA (SIGMA, Australia) was added and cells were grown for 2, 4, 6, 8, 10, 12 and 14 days before fixing and staining with Sulforhodamine B. Cells were visualized by phase contrast microscopy on corresponding days using an Olympus BX40 microscope and photographed using a DigitalSight DSL1 (Nikon) camera. Each experiment was performed on three separate occasions in quadruplicate. Heat treatment was performed at 95°C for 10 minutes. Pepsin digestion was performed with 1.5 mg bLALBA in HCl (pH 2) 1:500 enzyme to substrate for 60, 90 or 120 minutes/37°C. Products were purified using 1 kD Centrifugal device (Amicon Ultra). Products of digestion were analyzed on SDS-page gels.

### In situ detection of apoptosis

Mouse mammary epithelial cells, HC11, (1 × 10^5 ^cells per glass chamber slide) were plated and allowed to grow overnight in growth media. The following day media was removed and cells were treated in duplicate with 1 mg/ml bLALBA in growth media for 1, 2 and 4 hours or 0 mg/ml bLALBA. Apoptotic cells were detected using Apotag Peroxidase *in situ *Apoptosis Detection kit (Chemicon, Australia), according to the manufacturer's instructions for staining of cultured cells. Slides were counterstained with 0.5% methyl green and mounted using aqueous mounting medium. The apoptotic cells (brown staining) were viewed and counted using an Olympus BX50 microscope for light microscopy. The apoptotic index was defined by the percentage of brown (dark) cells among the total number of cells in each sample. For each treatment five to eight random fields were used to count apoptotic and live cells. A total of 711 cells (0 mg/ml LALBA), 587 cells (1 mg/ml bLALBA/1 hour), 473 (1 mg/ml bLALBA/2 hours) and 434 cells (1 mg/ml bLALBA/4 hours) were counted for each treatment. Results are represented as percentage of apoptotic and live cells.

### Statistics

For expression analysis unpaired student t-tests using 6 degrees of freedom were used to determine statistical significance. For apoptotic analysis a 2-tailed student *t*-test was used. Each test incorporated data from two independent experiments performed in duplicate on separate days.

## Authors' contributions

JAS constructed cDNA library, performed expression/promoter and translation analysis, proliferation assays, apoptotic assay, morphological analysis and drafted the manuscript. CL performed bioinformatics for annotation of sequences. KRN conceived of the study, and participated in its design and coordination. All authors read and approved the final manuscript.

## Supplementary Material

Additional file 1 **Table**1. PCR primer sequences. Click here for file
