# Supplementary material for: Lack of functional alpha-lactalbumin prevents involution in Cape fur seals and identifies the protein as an apoptotic milk factor in mammary gland involution
Source: BMC Biol. 2008 Nov 6;6:48. doi: 10.1186/1741-7007-6-48 (PMC2600633; doi:10.1186/1741-7007-6-48)
Supplement: Additional file 1 — Table1. PCR primer sequences. [file 1741-7007-6-48-S1.doc]

# Supplementary Methods Table 1. PCR primer sequences

**Primer Sequence**

**Cape fur seal:**

α-casein Forward 5’ CCCTACAAGCCTTTCTGCTG 3’

α-casein Reverse 5’ TGCTATCAGGATGCCAAGTG 3’

β-casein Forward 5’ CCCTACAAGCCTTTCTGCTG 3’

β-casein Reverse 5’ TGCTATCAGGATGCCAAGTG 3’

αS1-casein Forward 5’ TGCCGTGAAATTCCAAGAGA 3’

αS1-casein Reverse 5’ CCAAGCAGCAAAGGGATAAG 3’

αS2-casein Forward 5’ CCTGCTTTGGTTTGACTGGA 3’

αS2-casein Reverse 5’ TTTGCTTTGGCTCAGGATTG 3’

β-lactoglobulin-I Forward 5’ GAGATTAGACCCAGAGCCGAG 3’

β-lactoglobulin-I Reverse 5’ CGCGTCGAGCACAAAGAT 3’

α-lactalbumin (Full length) Forward 5’CAAAATGATGTCCTTTGTCT 3

α-lactalbumin (Full length) Reverse 5’ acaggagatgtcacagatc 3’

α-lactalbumin Forward 5’ GTCTCTCTGCTCCTGGTTGG 3’

α-lactalbumin Reverse 5’ TCTGTGCTGCCATTGTTACTG 3’

α-lactalbumin (promoter) Forward 5’ GTGGTGGTTAGGGGTGGAGAG 3’

α-lactalbumin (promoter) Reverse 5’CCTGGGACAGCTCACATTTT 3’

GAPDH Forward 5’ TGTGATGCTTGTGCTGAGTAT 3’

GAPDH Reverse 5’ TGAGGGTCTTGAGGGAGTTG 3’

**Mouse:**

α-lactalbumin Forward 5’ CGTTCCTTTGTTCCTGGTGT 3’

α-lactalbumin Reverse 5’ TACTCTGTGCTGCCGTTGTC 3’

GAPDH Forward 5’ AGGAGCGAGACCCCACTAAC 3’

GAPDH Reverse 5’ GTGGTTCACACCCATCACAA 3’

**Pig:**

α-lactalbumin Forward 5’ CAGGTGCTGAAAGACATGGA 3’

α-lactalbumin Reverse 5’ GTCACAGGAGATGCCACAGA 3’

GAPDH Forward 5’ GATGGTGAAGGTCGGAGTG 3’

GAPDH Reverse 5’ GGAAGATGGTGATGGGATTT 3’

**Tammar Wallaby:**

α-lactalbumin Forward 5’ GACTCATGACTACAGTCCATGCCAT 3’

α-lactalbumin Reverse 5’ GGACATGTAGCCATGAGGTCCAC 3’

GAPDH Forward 5’ GACTGATGACTACAGTCCATGCCAT 3’

GAPDH Reverse 5’ GTAAGCTTCCATGGAGAAATGGCCTCA 3’
